# The regulation of mitochondrial dynamics in neurite outgrowth by retinoic acid receptor β signaling

**DOI:** 10.1096/fj.201802097R

**Published:** 2019-03-11

**Authors:** Diogo Trigo, Maria B. Goncalves, Jonathan P. T. Corcoran

**Affiliations:** The Wolfson Centre for Age-Related Diseases, King’s College London, London, United Kingdom

**Keywords:** neuron, retinoid, growth cone, energy metabolism

## Abstract

Neuronal regeneration is a highly energy-demanding process that greatly relies on axonal mitochondrial transport to meet the enhanced metabolic requirements. Mature neurons typically fail to regenerate after injury, partly because of mitochondrial motility and energy deficits in injured axons. Retinoic acid receptor (RAR)-β signaling is involved in axonal and neurite regeneration. Here we investigate the effect of RAR-β signaling on mitochondrial trafficking during neurite outgrowth and find that it enhances their proliferation, speed, and movement toward the growing end of the neuron *via* hypoxia-inducible factor 1α signaling. We also show that RAR-β signaling promotes the binding of the mitochondria to the anchoring protein, glucose-related protein 75, at the growing tip of neurite, thus allowing them to provide energy and metabolic roles required for neurite outgrowth.—Trigo, D., Goncalves, M. B., Corcoran, J. P. T. The regulation of mitochondrial dynamics in neurite outgrowth by retinoic acid receptor β signaling.

Mitochondria are cellular organelles whose main functions are to generate ATP, buffer cytosolic calcium, and generate reactive oxygen species. Mitochondria also play an important role in the mechanism of axon extension, regeneration, and axon branching (1, 2). The elongating axon requires a continuous supply of energy in areas distal from the cell body (1); proper mitochondrial distribution is required for axonal regeneration (3), and indeed mitochondrial movement is increased following axonal injury (4).

This transport is regulated by various signaling pathways to efficiently respond to cellular needs and external factors. One of these pathways is hypoxia-inducible factor 1α (HIF-1α). Induced by decreased oxygen tension, the HIF-1α signaling pathway is traditionally associated with a first-level cell response to hypoxia, namely, by regulating mitochondrial recruitment, activity, and oxygen consumption (5, 6). Besides regulating mitochondrial activity, HIF-1α induces the expression of the modulator of mitochondrial movement, hypoxia up-regulated mitochondrial movement regulator (HUMMR), which promotes mitochondrial movement toward the distal part of neurites and synapses (7) by interacting with the transport enzymes mitochondrial Rho GTPase (MIRO) 1 and 2 (8, 9). Additionally, the mitochondrial stress chaperone glucose-related protein 75 (GRP75), a mediator of endoplasmic reticulum (ER)–mitochondrial coupling, has recently been associated with HIF-1α–mediated mitochondrial response to hypoxia (10–12).

It has previously been shown, in refs. 13–15, that retinoic acid induces axonal and neurite outgrowth by activating the retinoic acid receptor (RAR)-β, but the role of retinoids in mitochondrial mobilization or recruitment is yet to be described. Furthermore, considering the essential role of mitochondria in neurite elongation, and taking into account the role of hypoxia signaling in peripheral neurite regeneration, it is pertinent to investigate whether retinoic acid promotes neurite elongation in the CNS by acting *via* this signaling pathway.

Here we show that in cultured cortical neurons, RAR-β, in parallel to promoting neurite outgrowth, promotes mitochondrial membrane depolarization in the soma and its anterograde transport and proliferation along the neurite by activating the hypoxia signaling pathway. We describe that HIF-1α is required for both retinoid-induced neurite elongation and mitochondrion regulation, and we additionally show that RAR-β activation promotes the accumulation of mitochondria in the growing neurite. This is accomplished by facilitating the interaction of mitochondria with the chaperone GRP75, possibly by mediating mitochondrion-ER interaction.

## MATERIALS AND METHODS

### Primary neuronal cell cultures

Mouse primary cortical neurons were prepared as previously described in ref. 16. Cells were plated onto 5 μg/ml poly-d-lysine-coated 24-well cell culture plates, 75-cm^2^ flasks, or 35-mm glass-bottom culture dishes (MatTek, Ashland, MA, USA), depending on the experiment, at a density of 15 × 10^4^ cells per ml. Cells were cultured in neurobasal medium (Thermo Fisher Scientific, Waltham, MA, USA) containing 2% B27 serum-free supplement, 2 mM l-glutamine, 1.5% glucose, penicillin (100 U/ml), and streptomycin (100 g/ml), incubated at 37°C in a humidified atmosphere of 5% CO_2_ and 95% air. Cultures were 98% neurons, judged by βIII-tubulin staining. Unless mentioned otherwise, mouse primary cortical cultures were treated with 100 nM CD2019 (synthesized by Sygnature Chemical Services, Nottingham, United Kingdom) or vehicle (DMSO 0.1%, v/v) for 72 h. CD2019 is a RAR-β agonist capable of inducing axonal outgrowth in central primary cultures with a 5-fold and 12-fold selectivities for RAR-β over RAR-α and RAR-γ, respectively (16). The dose and treatment duration were based on our previous *in vivo* studies on activation of RAR-β signaling in the adult rat brain. HIF-1α was inhibited with CAY10585 (20 μm; Cayman Chemical, Ann Arbor, MI, USA), an (aryloxyacetylamino)benzoic acid analog that was determined by a reporter assay to inhibit HIF-1α protein accumulation and its target gene expression under hypoxic conditions, without altering HIF-1β levels (17).

### Immunocytochemistry

Immunocytochemistry was performed as previously described in ref. 16. Cortical neuron cultures were washed with PBS for 1 min, fixed in 4% paraformaldehyde for 20 min, washed 3 times for 5 min each in PBS, and permealized with 0.1% Triton X-100 in PBS for 4 min prior to being incubated in primary antibody in PBS with 0.02% Tween (PBS-T) overnight. Primary antibody was removed by washing 3 times for 5 min each in PBS-T; cultures were then incubated in the secondary antibody for 1 h at room temperature in PBS-T. The coverslips were then mounted using FluorSave reagent (Merck, Darmdstadt, Germany). Antibodies used were as follows: mouse monoclonal anti–βIII-tubulin (1:1000 for immunocytochemistry, against peptide EAQGPK; Promega, Madison, WI, USA), mouse monoclonal anti–HIF-1α (1:500, H1α67, aa 400–550; Abcam, Cambridge, MA, USA), mouse monoclonal anti-GRP75 (1:100, ab2799; Abcam), mouse monoclonal anti-actin (1:5000, AC-15; MilliporeSigma, Burlington, MA, USA), and Alexa Fluor 488 phalloidin (1:40, A12379; Thermo Fisher Scientific). Secondary antibodies for immunohistochemistry were Alexa Fluor 594 and Alexa Fluor 488 (1: 1000; Thermo Fisher Scientific). Hoechst stain was used to stain nuclei (1:10000; Thermo Fisher Scientific). Secondary antibodies for Western blotting were Alexa Fluor 680 and Alexa Fluor 800 (1:5000; Thermo Fisher Scientific). ER was stained with Cytopainter ER Staining Kit, Green Fluorescence (1:1000, ab139481; Abcam), according to the manufacturer’s instructions.

Imaging of mitochondria was performed by dyeing cells according to the manufacturer’s instructions with mitotracker red (500 nM; Thermo Fisher Scientific) for 30 min prior to fixation. Alternatively, cells were loaded with 20 nM tetramethylrhodamine, methyl ester (TMRM, T668; Thermo Fisher Scientific) for 45 min, prior to being placed in an incubator attached to a confocal microscope. TMRM is a cell-permeant fluorescent dye, sequestered by active mitochondria.

### Confocal microscopy

Multichannel fluorescence images were captured using a Zeiss LSM 700 laser-scanning confocal microscope (Carl Zeiss, Oberkochen, Germany) with a ×63 oil-immersion Aprochromat objective with an image size of 512 × 512 pixels, with a pinhole aperture of 1 Airy unit. Settings for gain, contrast, and brightness were optimized initially and held constant throughout each study so that all sections were digitized under the same conditions.

For colocalization studies, *z* stacks of the whole imaged neuron were taken (serial scans at different focal planes with a separation optimized by the software) as previously described in ref. 18. For time-lapse imaging analysis, TMRM loaded cells were imaged in a time series of 100 images 10 s apart. The Fiji distribution (*https://fiji.sc/*) of ImageJ software (National Institutes of Health, Bethesda, MD, USA) was used to measure pixel signal intensity and colocalization studies.

For neurite outgrowth assays, treated primary cortical neuron cultures were fixed in 4% paraformaldehyde, permeabilized with 0.1% Triton X-100 for 4 min, and stained with Alexa Fluor 488 phalloidin, or alternatively with the neuron-specific anti–βIII-tubulin. These were then imaged by confocal microscopy, and ImageJ-Fiji software was used to measure the length of the longest neurite in each neuron.

### Transfection of cortical neurons

GRP75 small interfering RNA (siRNA) (sc-35521; Santa Cruz Biotechnology, Dallas, TX, USA) and Lipofectamine 2000 transfection reagent (Thermo Fisher Scientific) in OptiMem (2% v/v) were prepared and allowed to stand for 5 min. They were then mixed and incubated at room temperature for 20 min before addition to cortical neurons plated in glass bottom dishes or 6-well plates, in a total volume of 1 ml, or in 24-well plates, in a total volume of 250 μl, with a final siRNA concentration of 10 nM. Cells were incubated for 7 h before adding an equal volume of supplemented neurobasal medium and incubated for additional 24 h, following which the medium was replaced with fresh supplemented neurobasal medium. Cells were incubated for an additional period of 24 h.

### Western blotting

Proteins were separated by SDS-PAGE on 10% (w/v) polyacrylamide gels and then transferred to a 0.45-μm pore size nitrocellulose membrane (BA85; GE Healthcare, Waukesha, WI, USA) using a Trans-Blot SD Semi-Dry Transfer Cell (Bio-Rad, Hercules, CA, USA). Nitrocellulose membranes were incubated in blocking solution consisting of 5% (w/v) skimmed milk powder in PBS-T (0.1%, w/v) for 1 h at room temperature, followed by incubation with the appropriate primary antibody diluted in blocking solution overnight at 4°C. Membranes were washed 3 times in PBS-T and incubated with species-specific secondary antibodies in blocking solution for 2 h at room temperature in the dark, after which the membranes were washed as above. Protein levels were corrected for loading differences by normalizing against β-actin levels. Proteins were detected by scanning at 700 and 800 nm using the Odyssey Detection System (Li-Cor Biosciences, Lincoln, NE, USA).

### Quantitative analysis

Signal intensity was analyzed by defining regions of interest with the actin or phalloidin staining and measuring the signal mean or integrated density with ImageJ-Fiji (19). Kymographs were plotted and analyzed using the Velocity Measurement Tool of ImageJ-Fiji. Colocalization was assessed by the Pearson product-moment correlation coefficient (Pearson’s *r* value) (20), obtained from the Coloc 2 plugin for ImageJ-Fiji. Fusion and fission events were counted individually in each time series, using the 3D Project Tool to obtain 3-dimensional reconstructions of *z* stacks of the neurite to distinguish fusion or fission events from overlapping mitochondria or mitochondria traveling together. Quantified anti–HIF-1α signals were normalized to a background area of 25 × 25 μm from each imaging field.

### Statistical analysis

Statistical studies were performed using Prism 5 for Mac OS X and Prism 6 for Windows (GraphPad Software, La Jolla, CA, USA). Unless stated otherwise, data were analyzed using nondirectional, 2-tailed, 2-sample, equal variance Student’s *t* test. Comparisons were made between appropriate groups, with α = 0.05.

## RESULTS

### RAR-β recruits mitochondria to the growing neurite

**Figure 1:**
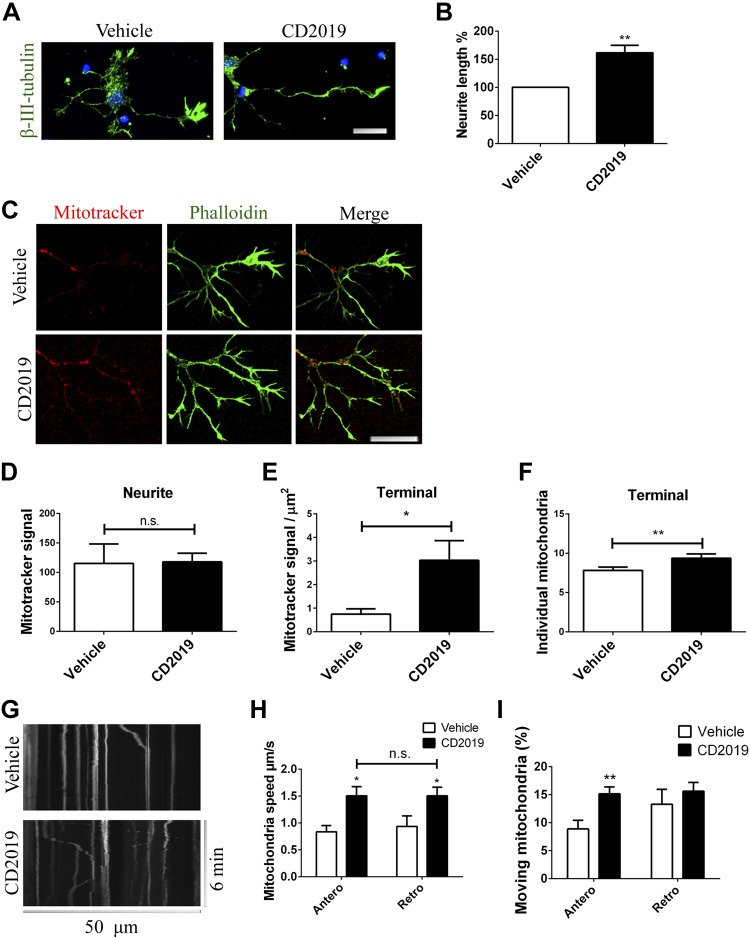
RAR-β activation promotes mitochondrial transport and accumulation toward the neurite terminal. *A*, *B*) Imaging (nuclear stain in blue; scale bar, 20 μm) (*A*) and quantification (*B*) of neurite outgrowth in neurons treated with either vehicle or CD2019 for 3 d. At least 5 neurons were quantified from each of at least 6 slides per condition. *C–E*) CD2019 compared with vehicle treatment resulted in an increase in the length of the longest neurite (scale bar, 10 μm) (*C*), accompanied by an increase of more than 3-fold in signal of mitotracker red in the neurites most distal 20 μm^2^ (terminal) (*D)*, with no differences in total mitochondrial presence in the whole neurite (*E*). At least 5 neurons were quantified from each of at least 6 slides per condition. *F*) This increase in mitotracker red signal is also supported by an increase in mitochondrial presence in the neurites most distal 20 μm^2^. *G*) Examples of kymographs recorded from neurons to determine the direction and speed of moving mitochondria of live neurons treated with either vehicle or CD2019, at least 30 min prior to imaging. At least 5 neurons were quantified from 5 (vehicle) and 8 (CD2019) slides. *H*, *I*) Mitochondria in neurons treated with agonist show an increase in the speed of anterograde (antero) and retrograde (retro) movement (*H*), and although the percentage of mitochondria moving retrogradely is not altered, the percentage of those moving anterogradely is increased by the agonist (*I*). N.s., not significant. *P < 0.05, **P < 0.005.

Given the high energy requirements of neurite outgrowth, we first asked whether activating RAR-β, required for neurite outgrowth in the CNS, was reflected in mitochondrial behavior in the neurite. Treatment of neuronal cultures with the RAR-β agonist CD2019 (100 nM for 72 h) results in neurite elongation (54% ± 32, *P* = 0.0018) (**Fig. 1*A*, *B***). We next assessed mitochondrial distribution along those neurites. Imaging of neurons stained with the mitochondrion dye mitotracker red (Fig. 1*C*) showed no statistically significant differences in mitotracker red signal on the cell body or the whole neurite between the RAR-β agonist– and vehicle-treated neurons. However, comparing the intensity of mitotracker red signal in the distal 20 μm^2^ of the neurite revealed a statistically significant increase in mitotracker red signal (0.75 ± 0.4 to 3.03 ± 1.6, *P* = 0.038). This increase was more significant when the number of mitochondria in the neurite terminal was tallied individually (7.8 ± 0.4 to 9.4 ± 0.6, *P* = 0.0044), indicating that this increase in mitotracker red signal results from an elevated mitochondrial presence in the growing neurite following RAR-β activation (Fig. 1*D*–*F*).

This population increment could result either from increased recruitment and transport toward the neurite or from greater mitochondrial proliferation in this region.

Concerning the former, imaging of live neurons loaded with TMRM (20 nM) revealed that acute activation of RAR-β (treated for 30 min prior to imaging with agonist, 100 nM) increases the speed of movement of healthy mitochondria, both in anterograde (*P* = 0.019) and retrograde directions (*P* = 0.048). However, even though the speed of moving mitochondria appears to also be increased independently of direction of transport, RAR-β activation increases the percentage of mitochondria moving anterogradely from 8.9 ± 3.5% to 15.2 ± 3.7% (*P* = 0.009), not altering the percentage of mitochondria moving toward the soma (*P* = 0.43) (Fig. 1*G*–*I*).

**Figure 2:**
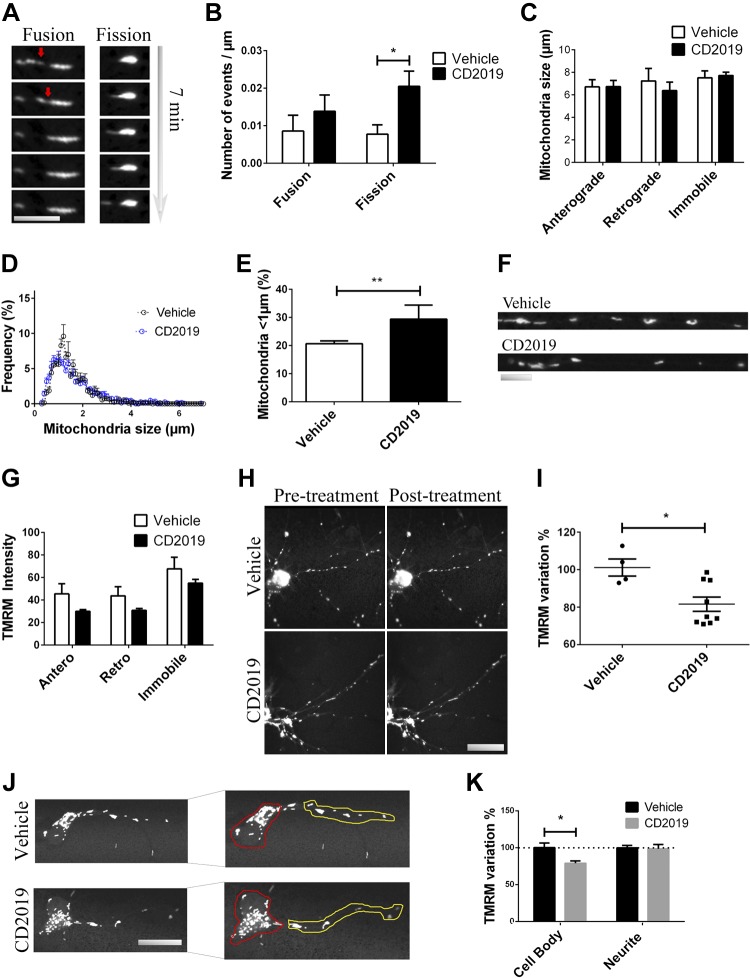
Mitochondrial transport and dynamics on the neurite is regulated by RAR-β. *A–C*) Acute treatment with CD2019 compared with vehicle (at least 30 min prior to imaging) alters mitochondrial dynamics, specifically increasing the number of fission events—imaging (scale bar, 5 μm) (*A*), quantification (*B*)—but the mean size of moving or stationary mitochondria in the neurite remains unaltered (*C*). *D*, *E*) However, analysis of the distribution of mitochondrial size frequency showed that treatment with CD2019 increased the number of mitochondria shorter than 1 μm long: imaging (*D*), quantification (*E*). *F–I*) Measurement of TMRM signal intensity in the neurite of live neurons shows that treatment with the agonist decreases the intensity of TMRM signal—imaging (scale bar, 5 μm) (*F*), quantification (*G*)—which is confirmed when mitochondrial TMRM signal in the whole neuron is analyzed, with treatment with CD2019 for 30 min causing a decrease of 20%: imaging (scale bar, 20 μm) (*H*), quantification (*I*). *J*, *K*) This reflects an increase in mean TMRM intensity signal in the cell body (red-delimited region), because the mean TMRM signal in the neurite (yellow-delimited region) remained unaltered following RAR-β activation: imaging (scale bar, 20 μm) (*J*), quantification (*K*). At least 5 neurons were quantified from each of at least 6 slides per condition. *P < 0.05, **P < 0.005.

As for the latter, even though comparing the mean length of mitochondria in the neurite revealed no differences, irrespective of their movement status (*P* = 0.99 for anterograde-moving, *P* = 0.54 for retrograde-moving, and *P* = 0.74 for immobile mitochondria), analysis of mitochondrial size distribution showed a significant increase in smaller mitochondria, particularly <1 µm, from 20.6 ± 1.0% to 29.3 ± 5.0% (*P* = 0.0026). Moreover, neuronal activation of RAR-β affected mitochondrial dynamics, substantiated by the increase in number of mitochondrial fission events, from 0.0077 ± 0.006 per micrometer in vehicle-treated neurons to 0.0204 ± 0.012 per micrometer in agonist-treated neurons (*P* = 0.035) (**Fig. 2*A*–*E***). Increased fission is a behavior associated with mitochondrial biogenesis (21) and previously observed in regenerating axons (22, 23).

Having identified that RAR-β activation recruits mitochondria to the growing neurite tip, we then analyzed its effect in mitochondrial activity. This was accomplished by measuring mitochondrial membrane potential following treatment with the RAR-β agonist, registering the intensity of TMRM signal in the neurite of live cortical neurons in culture. Acute activation of RAR-β (100 nM treatment 30 min prior to imaging) suggests a decrease in mitochondrial membrane potential in the neurite (*P* = 0.13 for anterograde-moving, *P* = 0.16 for retrograde-moving, and *P* = 0.29 for immobile mitochondria). This decrease in mitochondrial membrane potential was confirmed to be a statistically significant 20% decrease in TMRM integrated density signal when all the mitochondria in the neuron were taken into account (*P* = 0.012), suggesting that this decrease in membrane potential is more focused on the cell body. This was indeed confirmed when quantification of mean TMRM signal discriminated between cell body, where a 22% decrease in mean TMRM signal intensity was observed (*P* = 0.019), and the neurite, with mean TMRM signal intensity remaining unaltered (*P* = 0.819) (Fig. 2*F*–*K*).

### RAR-β signaling regulates HIF-1α expression

**Figure 3:**
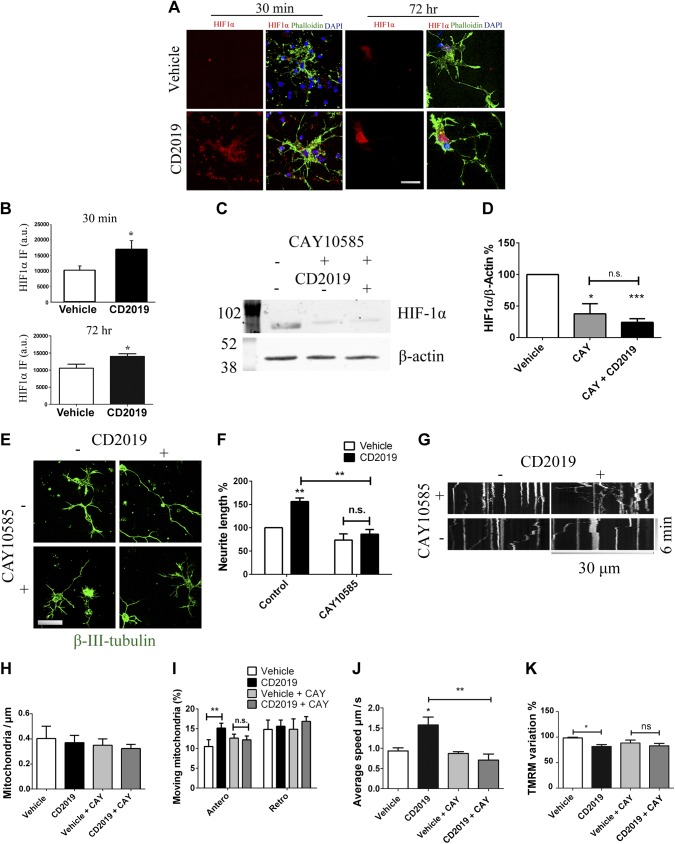
HIF-1α is involved in regulation of mitochondrial dynamics by RAR-β. *A*, *B*) Neuronal cultures treated with CD2019 for 30 min show an increase in the levels of HIF-1α, which was maintained when treatment was extended for 3 d compared with vehicle-treated cultures: imaging (nuclear stain in blue; green signal is phalloidin; scale bar, 10 μm) (*A*), quantification (*B*). Ten neurons were quantified from each of at least 4 slides per condition. *C*, *D*) Western blot of cell lysates of cortical primary cultures treated with vehicle, CAY15085, or CD2019 for 3 d shows that CAY15085 alone decreases the levels of HIF-1α which is not rescued by CD2019: imaging (*C*), quantification, (*D*). Western blots were repeated 3 times. *E*, *F*) Imaging (green signal is βIII-tubulin; scale bar, 20 μm) (*E*) and analysis (*F*) of neurons treated with CAY10585, CD2019, or both for 3 d. CAY10585 prevents the neurite outgrowth induced by CD2019. 10 neurons were quantified from each of at least 3 slides per condition. *G*–*J*) Acute treatment with CD2019 30 min prior to imaging (*G*) showed that inhibition of HIF-1α by CAY15085 does not alter mitochondrial presence in the neurite (*H*) or retrograde movement but does prevent the increases in anterograde-moving mitochondria (*I*) and speed of mitochondria (*J*) observed in the presence of CD2019. At least 5 neurons were quantified from each of at least 6 slides per condition. *K*) Moreover, the decrease in mitochondrial membrane potential, measured by TMRM following RAR-β agonist treatment, does not occur in the presence of the HIF-1α inhibitor CAY15085. At least 5 neurons were quantified from each of at least 6 slides per condition. CAY, CAY15085; n.s., not significant. *P < 0.05; **P < 0.005; ***P < 0.001.

Taking into account that activation of the hypoxia signaling pathway promotes axonal regeneration, we next asked if this pathway was regulated by RAR-β signaling. Given that HIF-1α can be regulated by retinoids by nontranscriptional mechanisms (24) and that we see an effect of RAR-β on mitochondrial function after 30 min, we looked at both acute (30 min) and chronic (72 h) CD2019 treatment on HIF-1α expression. At both time points, RAR-β activation results in an increase in HIF-1α expression by immunochemistry compared with control cultures: 65% increase at 30 min (*P* = 0.036) and 33% increase at 72 h (*P* = 0.049) (**Fig. 3*A*, *B***).

These observations, together with the data showing mitochondrial recruitment to the neurite terminal, suggests that RAR-β promotes mitochondrial recruitment toward the growing neurite, where energy production is promoted *via* increased oxidative phosphorylation, in a HIF-1α–mediated process. Having established the importance of HIF-1α signaling in neurite outgrowth, we further studied its effect by inhibiting HIF-1α in cultured neurons with CAY10585 (10 μg/ml), which inhibits HIF-1α accumulation in a concentration and time-dependent manner and significantly suppresses transcriptional activity of HIF-1α target genes, obtaining complete inhibition at similar concentrations (17). Neurons were treated for 72 h, which decreased the presence of HIF-1α in lysates to 37% of that in control-treated lysates (*P* = 0.018), in a manner not rescuable by CD2019, which had a similar decrease in HIF-1α levels to 24% of control (*P* = 0.0002) (Fig. 3*C*, *D*). In the CD2019 plus CAY10585–treated cultures, the RAR-β–mediated neurite outgrowth was also inhibited, with the mean length of the longer neurite being comparable to the length seen in vehicle-treated cultures (1-way ANOVA followed by Tukey’s test, *P* < 0.005).

Considering that RAR-β activation has an effect in mitostasis regulation, as early as after 30 min of treatment, we next studied the role of HIF-1α in this regulation. Inhibition of HIF-1α does not alter the total percentage of mitochondria moving in the neurite in live neurons (*P* = 0.48) but prevents the increase in speed of moving mitochondria observed with acute activation of RAR-β, decreasing the mean speed of moving mitochondria to that of vehicle-treated neurons (1-way ANOVA followed by Tukey’s test, *P* < 0.005). While not altering mitochondrial presence in the neurite or the percentage of mitochondria moving in either direction, when compared with control, the increase in anterograde-moving mitochondria observed with activation of RAR-β was prevented with HIF-1α inhibition, not altering the percentage of retrograde-moving mitochondria. Additionally, the decrease in TMRM signal intensity observed following activation of RAR-β is also absent when HIF-1α is inhibited (Fig. 3*E*–*K*).

### Mitochondrial interaction with GRP75 is required for neurite elongation

The mitochondrion-associated stress chaperone GRP75 has recently been described to play a role in neuronal ER–mitochondrial coupling and sensitivity to oxidative stress and hypoxia, in a process involving HIF-1α (10, 25). Because GR75 is highly linked with mitochondrial response to hypoxia and oxygen limitation (11, 12), and considering that it has been shown to bind RARs (26), we next looked at its expression levels to further clarify its role in mediating the process of mitochondrial recruitment to the growing neurite.

**Figure 4:**
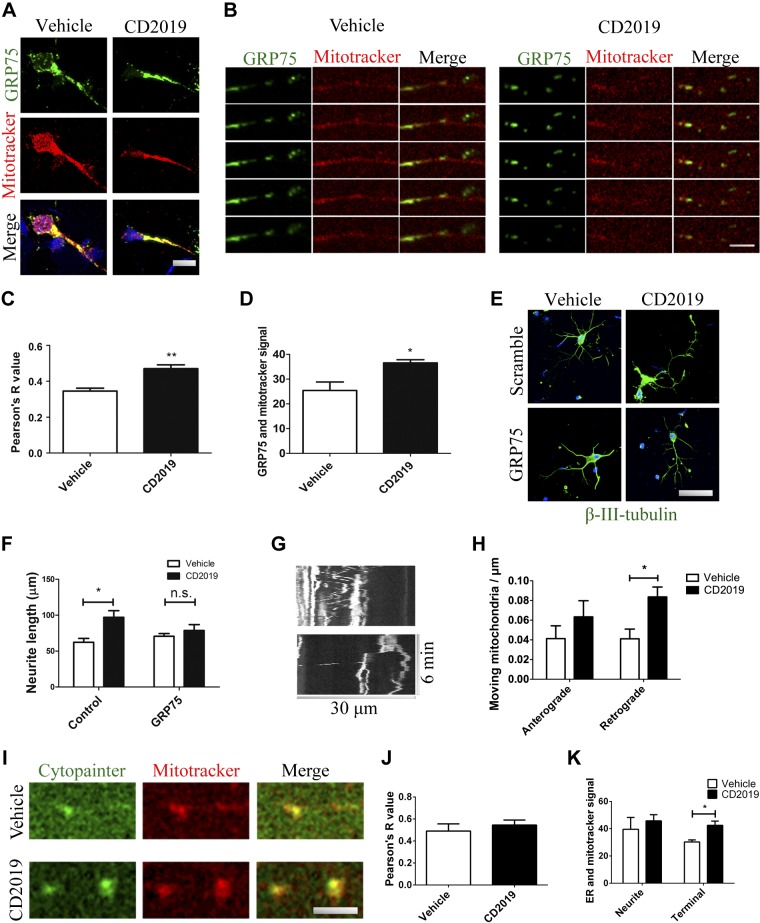
In the growing neurite, mitochondria interact with GRP75 and the ER following RAR-β activation. *A*–*D*) Neurons treated with CD2019 for 3 d [imaging of cell body and neuron (scale bar, 10 μm) (*A*); zoomed-in imaging of the neurite in a series of *z*-stack slices (scale bar, 20 μm) (*B*)] show an increase in colocalization between GRP75 and mitotracker red signals compared with vehicle-treated neurons (*C*, *D*). *E*, *F*). Treatment of neurons with siRNA for GRP75 negated the effect of neurite elongation of treatment with RAR-β agonist: imaging (green signal is βIII-tubulin; scale bar, 20 μm) (*E*), quantification (*F*). *G*, *H*) Live imaging of neurons treated with GRP75 siRNA showed that acute treatment with RAR-β agonist (at least 30 min prior to imaging) doubled the number of mitochondria moving in the retrograde direction, whereas the number of anterograde-moving mitochondria is unaltered compared with vehicle-treated neurons: imaging (*G*), quantification (*H*). *I*, *J*) In similarly treated preparations, ER staining and mitotracker red staining (scale bar, 5 μm) (*I*) show a solid Pearson’s *r* = 0.5 (*J*), which is not altered significantly with activation of RAR-β. *K*) The neurite’s more distal 20 μm^2^ reveals a significant increase in the overlap between ER and mitochondria. At least 5 neurons were quantified from each of 3 slides per condition. N.s., not significant. *P < 0.05, **P < 0.005.

Immunostaining of cultured cortical neurons that had been treated with CD2019 (100 nM) or vehicle for 72 h shows that in the presence of the RAR-β agonist there is an increase in colocalization of GRP75 and the mitochondrion dye mitotracker red, suggesting that not only is GRP75 up-regulated in RAR-β activated neurites but its binding to mitochondria is also elevated, established by colocalization analysis. Indeed, RAR-β activation caused a 40% increase in GRP75 signal coincident with mitotracker red (*P* = 0.038), and colocalization between immunostained GRP75 and mitotracker red increased from 0.35 ± 0.03 in vehicle-treated neurons to 0.47 ± 0.04 (Pearson’s *r* value, *P* = 0.0091) (**Fig. 4*A*–*D***).

Having established that GRP75 and its interaction with mitochondria are up-regulated following RAR-β activation, we next silenced GRP75 with siRNA to explain its role in mitochondrial recruitment by RAR-β. Treatment with GRP75 siRNA was sufficient to ablate the effect of neurite elongation in primary cultures of cortical neurons following treatment with RAR-β agonist (100 nM for 72 h, 1-way ANOVA followed by Tukey’s test, *P* < 0.05). Live imaging of GRP75-silenced neurons additionally revealed that acute treatment with RAR-β agonist (30 min prior to imaging) causes a significant increase of 102% in the number of mitochondria moving in the retrograde direction (*P* = 0.039) (Fig. 4*E*–*H*). Having established that activation of RAR-β promotes GRP75 association with mitochondria and that this association is required to accumulate mitochondria in the growing neurite, and given that GRP75 mediates ER-mitochondrial interaction, we examined whether the association between the ER and mitochondria was similarly up-regulated by RAR-β activation.

Colocalization analysis of cortical primary cultures stained with Cytopainter ER and the mitochondrion dye mitotracker red shows a solid Pearson’s *r* = 0.6 between ER and mitochondria. The levels of coincident signal between ER and mitotracker red in the whole neuron were unaltered following RAR-β activation, but when this analysis was restricted to the distal 20 μm^2^ of the neurite, an increase of 40% (*P* = 0.025) was observed in agonist-treated neurons (Fig. 4*I*–*K*). These results suggest that, following RAR-β activation, mitochondria recruited to the growing terminal are coupled to the ER in an interaction regulated by GRP75.

## DISCUSSION

**Figure 5:**
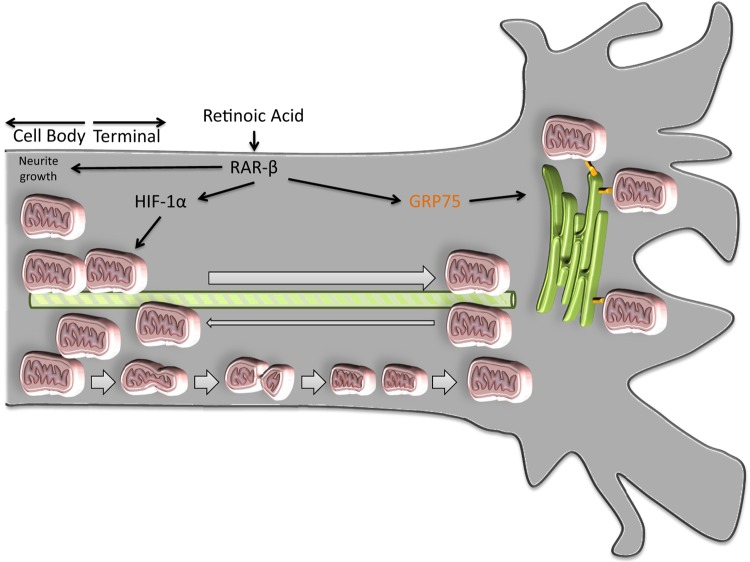
Model for mitochondrial recruitment by RAR-β in the growing neurite. Activation of RAR-β in cortical neuronal cultures promotes neurite elongation. This process involves, in part, up-regulation of HIF-1α signaling, which promotes mitochondrial membrane depolarization, decreasing oxidative phosphorylation in the cell body, but not neurite tip. Activation of RAR-β also causes a HIF-1α–dependent increase in mitochondrial proliferation and increases the speed of mitochondrial movement along the neurite. Following activation of RAR-β, GRP75 mediates mitochondrial binding to the ER in the terminal, anchoring the recruited mitochondria to this region, decreasing the number of mitochondria being transported retrogradely, and effectively increasing the mitochondrial presence in the growing neurite terminal.

In this study, we have observed that in primary cultures of cortical neurons, activation of RAR-β not only induces neurite outgrowth but also mobilizes mitochondria to the growing neurite (**Fig. 5**). These processes are mediated by HIF-1α and are accomplished by up-regulating mitochondrial presence in the tip of the growing neurite by increasing the speed and number of mitochondria moving anterogradely and by promoting mitochondrial proliferation. Recruited mitochondria accumulate in the growing terminal, where they closely associate with the chaperone GRP75 and the ER. In parallel, mitochondrial membrane potential is not statistically significantly decreased in the neurite but is evidently decreased when the cell body is considered. This suggests that mitochondrial membrane potential, and consequentially oxidative phosphorylation and energy production, could be favored in the growing neurite at the expense of the cell body. We observed no correlation between mitochondrial membrane potential and direction of transport in the neurites. Even though retrogradely moving mitochondria have been associated with depolarized membranes (27, 28), other studies found no distinction between anterograde and retrograde populations (29, 30), as was the case of our observations.

### Regulation of mitochondrial dynamics by RAR-β

Previous work has shown that activation of RAR-β stimulates neurite growth (13–15). Because the process of constant cytoskeletal rearrangement in the elongating axon requires a continuous supply of energy in areas distal from the cell body (31), proper mitochondrial distribution is required for axonal regeneration (3), and axonal injury increases mitochondrial movement (4). Therefore, for a pathway to promote axonal growth, it must also be capable of actively recruiting mitochondria to the growing axon.

We describe that RAR-β signaling–dependent growth is associated with anterograde mitochondrial movement, resulting in an increased presence of mitochondria in the neurite terminal. In large cells such as axons, the main reason for mitochondrial anterograde movement is typically to distribute new, healthy mitochondria (32) to sites of high energy demand, such as the node of Ranvier or the growing tip (33, 34).

We observe that activation of RAR-β in primary cultures of cortical neurons increases the number of submicrometer-long mitochondria in the neurite, resulting from an increase in mitochondrial fission, pinching off segments that result in daughter mitochondria. This process is associated with mitochondrial redistribution to regions of increased metabolic demand (30, 35), and no overall changes to the mean length of mitochondria were observed, independent of mitochondrial movement status. This modulation of mitochondrial dynamics occurring following RAR-β activation appears, then, to be related not to mitochondrial degradation but to increased mitochondrial proliferation (21) required for axonal growth (36).

### Recruitment and regulation of mitochondrial dynamics by the hypoxia signaling pathway

The neuronal process of mitochondrial transport occurs through different mechanisms, depending on the direction of movement (37), occurring through dynein motors in the case of retrograde transport (38), and kinesin motors in the case of anterograde transport (39). Even though the regulation mechanism of these motor proteins is not yet clear (40), the MIRO protein family is associated with mitochondrial axonal stopping (41, 42). This mechanism is particularly interesting, considering that MIRO acts by interacting with the hypoxia mediator HUMMR, a neuronal protein up-regulated in mitochondria *via* HIF-1α, which promotes anterograde transport (7).

Additionally, activation of RAR-β in cortical neuronal cultures decreases somal mitochondrial membrane potential, similar to what has been described in isolated mitochondria (43). In parallel to promoting neurite outgrowth, we have presented evidence that activation of RAR-β starts a signaling cascade that fulfills the heightened energy need in the neurite terminal. As a result, RAR-β activation appears to decrease oxidative phosphorylation in the cell body, reflected in a decrease in membrane potential, minimizing substrate usage and oxygen consumption away from the growing neurite, where energy requirement is higher.

Our data show that HIF-1α is up-regulated in cortical neuronal culture following RAR-β activation. We have shown that this can be *via* a nongenomic effect of RAR-β signaling, because it occurs as early as 30 min after agonist treatment. Nongenomic events of retinoid signaling are now emerging as important players in the maintenance and regeneration of the nervous system, and include activation of AKT (44), synapse transmission (45), and proliferation of stem cells, which is also due to increased stability of HIF-1α by retinoid signaling (46). In addition, chronic treatment with the RARβ agonist maintains HIF-1α expression, which suggests that RAR-β signaling may controls HIF-1α at the translational level as well as regulating its stability. HIF-1α expression is necessary for retinoid-mediated neurite outgrowth to occur, because inhibition of HIF-1α with the HIF-1α inhibitor CAY10585 prevents the increase in neurite length observed following RAR-β activation. Inhibition of HIF-1α also prevents the recruitment of mitochondria to the growing neurite, negating the increase in speed of mitochondrial movement observed following treatment with RAR-β agonist in live neurons. Moreover, inhibition of HIF-1α also prevents the decrease in mitochondrial membrane polarization observed in the cell body in response to RAR-β activation.

Besides promoting peripheral axonal regeneration, hypoxia-pathway signals are crucial in regulating mitochondrial dynamics: HIF-1α regulates mitochondrial oxygen consumption (47), and the HIF-1α–induced HUMMR interacts with mediators of mitochondrial transport (9), promoting anterograde mitochondrial movement (7). The HIF-1α–mediated increase in anterograde mitochondrial transport following RAR-β activation could occur using the same machinery. We show that HIF-1α modulates mitochondrial activity, promoting its motility and minimizing oxidative phosphorylation at the cell body. At the growing neurite, where membrane depolarization signals are absent, mitochondrial membrane repolarizes, promoting oxidative phosphorylation, by which mitochondria can generate the energy required for the process of neurite elongation.

### Mitochondrial accumulation in the growing neurite is dependent on the interaction with GRP75

The increase in mitochondrial movement throughout the neuron is stopped at the growing neurite terminal, where mitochondria are halted. If this process were only caused by the absence of promovement signals, a residual stochastic increase in retrograde transport should still presumably occur because of increased mitochondrial presence. Because such an increase was not observed, it follows that mitochondria recruited to the neurite must actively be maintained at this location.

The chaperone GRP75 is an essential mitochondrion-targeted chaperone (48, 49), up-regulated in stress situations such as starvation, oxidative stress or ischemia (50, 51), and, importantly, is involved in mitochondrial distribution (52).

We show here that RAR-β–mediated recruitment of mitochondria to the growing neurite is accompanied by an up-regulation in GRP75 and an increase in mitochondrial colocalization with GRP75. Although found in other subcellular locations, GRP75 was originally identified as a mitochondrial matrix-resident chaperone protein (53), and data suggest that its preferential location in the neurite is indeed associated with the mitochondria.

We further show that GRP75 is required for neurite outgrowth, because its silencing with siRNA ablates the effect of neurite elongation. Interestingly, silencing of GRP75 also resulted in a drastic increase in mitochondrial retrograde transport (*i.e.*, in the direction of the cell body) following activation of RAR-β. These results indicate that GRP75 is required to maintain recruited mitochondria at the neurite terminal.

In addition to its role in energy homeostasis, mitochondria coordinate with the ER, being physically tethered through the mitochondrion-associated ER membrane complex to regulate cellular levels of intracellular calcium (54), and the importance of ER in the axon has recently been characterized (55). Considering that the mitochondrion-ER interface is mediated by GRP75, which colocalizes with mitochondria and mitochondrion-associated ER membranes (56), the increase in mitochondrion-GRP75 colocalization following RAR-β activation raises the hypothesis that it could be up-regulating mitochondrion-ER interaction.

Indeed, RAR-β activation is accompanied by increased association between the ER and mitochondria in the distal end of the neurite, where mitochondrion-ER interaction is exacerbated. This interaction could be increased because of structural purposes (*i.e.*, anchoring mitochondria in specific neuronal locations) or functional reasons (such as calcium regulation), and GRP75 has long been described to determine ER-mitochondrial coupling, mediating the interaction of the mitochondrial voltage-dependent anion channel with the reticular inositol 1,4,5-triphosphate receptor (56).Therefore, mitochondria could not only be playing a crucial role generating chemical energy required for axonal and neurite outgrowth, but additionally helping control the calcium presence, an important regulator of axon outgrowth and guidance (57).

## Abbreviations

ER: endoplasmic reticulum
GRP75: glucose-related protein 75
HIF-1α: hypoxia-inducible factor 1α
HUMMR: hypoxia up-regulated mitochondrial movement regulator
MIRO: mitochondrial Rho GTPase
PBS-T: PBS with 0.02% Tween
RAR: retinoic acid receptor
siRNA: small interfering RNA
TMRM: tetramethylrhodamine, methyl ester
